# Supplementary material for: Curating models from BioModels: Developing a workflow for creating OMEX files
Source: PLoS One. 2024 Dec 5;19(12):e0314875. doi: 10.1371/journal.pone.0314875 (PMC11620473; doi:10.1371/journal.pone.0314875)
Supplement: S1 Table — (PDF) [file pone.0314875.s001.pdf]

S1 Table. Finding parameter values in papers.

We mostly referred to the SBML files for the model information. However, we also referred to the corresponding papers to validate, correct, and extend the models. Most parameter information was provided in the figure titles, however, we also found some valuable information in the main text and tables in the paper. Therefore, we suggest checking parameters following the order of SBML files, figure titles, text content, and tables in the papers.

Table S1. Finding parameter values in papers.

BioModel ID	Figure title	Text	Table	BioModel ID	Figure title	Text	Table
BIOMD0000000003				BIOMD0000000850	X		
BIOMD0000000005	X	X		BIOMD0000000877	X	X	
BIOMD0000000010				BIOMD0000000894			
BIOMD0000000079	X			BIOMD0000000909	X		
BIOMD0000000548	X			BIOMD0000000911			
BIOMD0000000552	X			BIOMD0000000916	X		
BIOMD0000000555				BIOMD0000000930	X		
BIOMD0000000618				BIOMD0000000932			
BIOMD0000000642				BIOMD0000000933			
BIOMD0000000667				BIOMD0000000939		X	
BIOMD0000000671				BIOMD0000000947			
BIOMD0000000704	X			BIOMD0000000948			
BIOMD0000000712				BIOMD0000000949	X		X
BIOMD0000000720	X			BIOMD0000000953			
BIOMD0000000745		X		BIOMD0000000964	X		
BIOMD0000000757	X	X		BIOMD0000000967			
BIOMD0000000780	X	X		BIOMD0000000970			
BIOMD0000000781	X	X		BIOMD0000000984		X	
BIOMD0000000782	X	X		BIOMD0000000986			
BIOMD0000000785	X			BIOMD0000001004			
BIOMD0000000793				BIOMD0000001006			
BIOMD0000000795				BIOMD0000001023			
BIOMD0000000799	X			BIOMD0000001026	X		
BIOMD0000000815	X			BIOMD0000001037			
BIOMD0000000839				BIOMD0000001038			

The BioModel ID, Figure title, Text, and Table columns are for the model IDs in the BioModels Database, the sentences in the figure title, the text content in the paper, and the tables in the paper.
