# Supplementary material for: Curating models from BioModels: Developing a workflow for creating OMEX files
Source: PLoS One. 2024 Dec 5;19(12):e0314875. doi: 10.1371/journal.pone.0314875 (PMC11620473; doi:10.1371/journal.pone.0314875)
Supplement: S1 Appendix — (PDF) [file pone.0314875.s002.pdf]

S1 Appendix. Non-reproduction examples with certain limitations from papers.

For BioModels 548, 642, 757, 780, 877, 949, and 984, there were non-reproductions due to given parameters. We were able to reproduce some of the figures in the paper, however, were not able to reproduce the other relevant figures by only adjusting the corresponding parameters provided. In detail, we were able to reproduce Fig 3 in BioModel 548, but were not able to reproduce Fig 2 by only adjusting the parameter m from 25 to 10. We were able to reproduce Fig 1 in the BioModel 642 with parameter $pi = 0$ but were not able to reproduce Fig 2 and Fig 3 with parameters $pi = 0.3$ or $pi = 0.6$. For BioModel 757, we successfully reproduced Fig 1, but couldn't reproduce Fig 2 and Fig 3 with the parameters given. For BioModel 780, we were able to reproduce Fig 6, but couldn't reproduce Fig 7 and Fig 8 with the parameters given. For BioModel 877, we were able to reproduce Fig 1 and Fig 2, but couldn't reproduce Fig 3 and Fig 4 with the parameters given. For BioModel 949, we were able to reproduce Fig 2, but couldn't reproduce Fig 3 with the parameters given. For BioModel 984, we were able to reproduce Fig 3, but couldn't reproduce Fig 4 and Fig 5 with two small values of parameter k.

For BioModel 005, Fig 3c was not reproducible due to the lack of formula of k_6. For BioModel 745, there were no data of tumor volume data given to reproduce Fig 5 and Fig 6. For BioModel 909, the data in Fig 11 and Fig 12 were not given. For BioModel 953, we were able to reproduce Fig 7B, but there were no clues regarding how to reproduce Fig 7C.
